# Sialyllactose Enhances the Short-Chain Fatty Acid Production and Barrier Function of Gut Epithelial Cells via Nonbifidogenic Modification of the Fecal Microbiome in Human Adults

**DOI:** 10.3390/microorganisms12020252

**Published:** 2024-01-25

**Authors:** Yohei Sato, Masaya Kanayama, Shiori Nakajima, Yukihiro Hishida, Yuta Watanabe

**Affiliations:** 1 Institute of Health Science, Kirin Holdings Co., Ltd., 2-26-1 Muraoka-Higashi, Fujisawa 251-8555, Japan; yohei_sato@kirin.co.jp (Y.S.); masaya_kanayama@kirin.co.jp (M.K.); yh0852145269@ymail.ne.jp (Y.H.); 2 Health Science Business Department, Kirin Holdings Co., Ltd., 4-10-2 Nakano, Tokyo 164-0001, Japan; shiori_nakajima@kirin.co.jp

**Keywords:** human milk oligosaccharides, sialyllactose, 3′-sialyllactose, 6′-sialyllactose, nonbifidogenic effect, *Bifidobacterium*, short-chain fatty acids, gut, microbiome

## Abstract

Although various benefits of human milk oligosaccharides (HMOs) have been reported, such as promoting *Bifidobacterium* growth in the infant gut, their effects on adults have not been fully studied. This study investigated the effects of two types of sialyllactose, 3′-sialyllactose (3′-SL) and 6′-sialyllactose (6′-SL), on the adult intestinal microbiome using the simulator of human intestinal microbial ecosystem (SHIME^®^), which can simulate human gastrointestinal conditions. HPLC metabolite analysis showed that sialyllactose (SL) supplementation increased the short-chain fatty acid content of SHIME culture broth. Moreover, 16S rRNA gene sequencing analysis revealed that SL promoted the growth of *Phascolarctobacterium* and *Lachnospiraceae*, short-chain fatty acid-producing bacteria, but not the growth of *Bifidobacterium*. Altogether, both types of SL stimulated an increase in short-chain fatty acids, including propionate and butyrate. Additionally, SHIME culture supernatant supplemented with SL improved the intestinal barrier function in Caco-2 cell monolayers. These results suggest that SL could act as a unique prebiotic among other HMOs with a nonbifidogenic effect, resulting in intestinal barrier protection.

## 1. Introduction

There are approximately 1000 microbial species and more than 10^13^ microbial cells in the human gut, which use the remaining components, such as amino acids and oligosaccharides, of the gut, forming the gut microbiota [1,2]. Recent research has shown that the gut microbiome contributes to human health by developing immune systems [3,4] and modulating metabolism [5]. Short-chain fatty acids (SCFAs), specifically produced by the gut microbiota, play various important roles in the gut health of the human host. SCFAs contribute to the acidic conditions in the gut and inhibit the growth of harmful bacteria, such as coliform or *Enterococcus*. In particular, butyrate maintains the integrity of the gut barrier as an energy source for intestinal epithelial cells (IECs) [6]. Inflammatory bowel disease (IBD) is triggered by lowered intestinal barrier function and the entry of toxins derived from the diet and gut microbiota into the body [7]. The gut microbiome of patients with IBD had lower diversity than that of healthy individuals and decreased bacteria producing butyrate, which resulted in worse inflammation [8]. Furthermore, it promotes metabolic cycles in IECs and controls gut immune responses by inducing regulatory T cells directly [9,10]. In this manner, the gut microbiome coexists with human cells and plays an important role in human physiology.

Human milk oligosaccharides (HMOs), the third most abundant solid nutrients in human breast milk after lactose and lipids, contribute to the development of the microbiome in infants. There are more than 200 types of HMOs, and each HMO appears to have a specific function due to its different structure based on its constitutive components of glucose, galactose, *N*-acetylglucosamine, fucose, and *N*-acetylneuraminic acid (sialic acid) [11,12]. Moreover, 2′-fucosyllactose (2′-FL), which is composed of fucose and lactose, promotes *Bifidobacterium* [13,14,15]. Consistently, the rate of *Bifidobacterium* in infants supplemented with 2′-FL increases compared to infants supplemented with formula milk without 2′-FL [16,17]. Although HMOs are known to support gut microbiome development during infancy, the bioavailability of HMOs is considered to vary among microorganisms. For example, some research revealed that *Bifidobacterium* species that possess extracellular fucosidase or both the ABC transporter and intracellular fucosidase utilize 2′-FL [14]. Therefore, the function of HMOs is considered to depend on the microorganisms in the gut. *Bifidobacterium* is not an effective target since it is minor in the adult gut. Targeting a common useful microorganism between the infant and the adult would be more effective in the function of HMOs. However, little is known about how HMOs, including 2′-FL, regulate other microorganisms’ proliferation.

As mentioned above, there are 1000 types of microorganisms in the human gut. In addition, the composition of the human gut microbiome is intricately regulated by the pH, oxygen concentration, temperature, and limited nutrients. To analyze the effect of nutrients on the gut microbiome, some types of in vitro methods have been developed. The simulator of human intestinal microbial ecosystem (SHIME^®^, Prodigest, Ghent, Belgium) is a multicompartment dynamic human gut simulator. In the SHIME model, programmatically controlled culture conditions (medium flow, pH, and N_2_ gas) allow the evaluation of various treatments on the microbiome in an environment similar to the human gut. In recent years, researchers have used the SHIME for the nonclinical evaluation of prebiotics and probiotics [18,19,20,21,22,23]. Bondue et al. reported that symbiotic treatment combining 3′-sialyllactose (3′-SL) and *Bifidobacterium crudilactis* showed a bifidogenic effect on the fecal microbiome of 1–2-year-old infants [22]. This is the first study to evaluate the effect of sialyllactose (SL) on the gut microbiome derived from adults with the SHIME model. Furthermore, we investigated the effects of bacterial metabolites in SHIME cultures on the human intestinal cell line. This study reveals the unique microbiome-modulating effects of SL, and it provides information on the maintenance and improvement of gut health in adults.

## 2. Materials and Methods

### 2.1. Chemical Reagents

All the chemicals were obtained from Fujifilm Wako Pure Chemical Corporation (Osaka, Japan) unless otherwise stated. The 3′-SL and 6′-sialyllactose (6′-SL) were obtained from Kyowa Hakko Bio Co., Ltd. (Tokyo, Japan). Both SLs were biosynthesized via bacterial fermentation.

### 2.2. Collection of Fecal Samples

The fecal sample collection and related experiment were conducted in accordance with the Declaration of Helsinki, and the protocol was approved by the Research Ethical Review Committee of Kirin Holdings Company, Limited (Kirin Holdings Company, Limited, Tokyo, Japan; file number 2019-002). All the subjects provided their informed consent for inclusion before they participated in the study. Fresh feces were collected from three healthy adult donors (more than 20 years old). All the donors met the following criteria: (1) understand the objective and process of this research and agree to participate in this research under their responsibility; (2) have a relative abundance ratio of the fecal microbiome profile based on the Mykinso service (Cykinso, Inc., Tokyo, Japan) described below; (i) ≥1.5% *Bifidobacterium*; (ii) ≥1% lactate-producing bacteria; (iii) ≥20% butyrate-producing bacteria; and (iv) ≥0.03% equal-producing bacteria; (3) have none of the following viruses: human immunodeficiency virus, hepatitis A virus, hepatitis B virus, hepatitis C virus, human T-cell leukemia virus type I; (4) not be treated by any antibiotics for 3 months until the fecal collection day; (5) not have any chronic disease; (6) not drunk alcohol one day before the fecal collection day; and (7) not pregnant or breastfeeding. The fecal samples were transported to our laboratory under an anaerobic atmosphere using an AnaeroPack system (Mitsubishi Gas Chemical, Tokyo, Japan) and an ice pack. The fecal samples were inoculated with SHIME in less than 12 h after defecation.

### 2.3. Culture of the Fecal Microbiome (SHIME)

**Figure 1:**
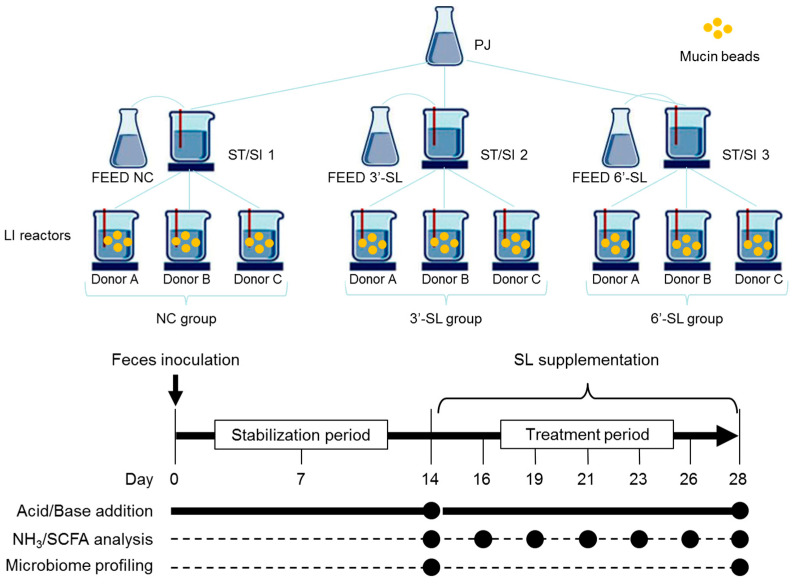
Experimental design of the SHIME.

Mucin bead-added SHIME (M-SHIME^®^) was used to conduct the present study. Based on the method of Šuligoj et al. [21], the M-SHIME setup was modified to use fecal samples collected from three donors (donors A, male; donor B, male; and donor C, male) and to combine three different conditions (nine conditions in total). A graphical abstract is shown in Figure 1. Briefly, a stomach/small intestinal reactor (ST/SI) was connected to three reactors that simulated the large intestine (LI). In contrast to the ST/SI reactors, which operated according to a fill-and-draw principle, the LI reactors had a fixed volume of 250 mL each. The pH of LI was controlled within a fixed interval (6.6–6.9) by adding 0.5 M HCl and 0.5 M NaOH. In addition to a luminal phase, each LI contained 18 mucin-coated microcosms prepared according to Van den Abbeele et al. [24] to allow simulation of the mucosal microbiota. Moreover, 50% of the mucin beads were renewed three times a week by opening the reactors. All the reactors were airtight, continuously agitated (300 rpm), and temperature controlled (37 °C). Anaerobiosis was maintained by flushing the headspace of all the reactors with nitrogen gas.

The experiment consisted of stabilization (days 0–14) and the SL treatment period (days 15–28). On day 0, the LI reactors of each of the nine units were filled with 250 mL of SHIME nutritional medium (PDNM002B, Prodigest) and inoculated with a fecal suspension (5% *v*/*v*) of one of the three donors investigated (Donor A, B or C), as prepared according to Moens et al. [25]. Each ST/SI reactor received three feeding cycles per day of 210 mL of SHIME nutrition medium (FEED) and 90 mL of bile/pancreatic juice (PJ, oxgall (Becton, Dickinson and Company, 6 g/L), sodium bicarbonate (12.5 g/L), and porcine pancreatin (0.9 g/L)). Then, the content of the reactor was pumped to the LI, and excess liquid was removed from the waste vessel. During the stabilization period (days 0–14), all the ST/SI reactors received the FEED without additional ingredients. During the treatment period (days 15–28), the FEED pumped into one of the three ST/SI was added 3′-SL (6 g/L), the FEED pumped into another ST/SI was added 6′-SL (6 g/L), and nothing was added to the remaining FEED as a negative control (NC). In summary, 9 LI conditions were performed to combine three donors (A, B, and C) and three conditions of FEED (NC, 3′-SL, and 6′-SL). The culture broth was sampled in the LI reactors every 2 or 3 days after the 9th day of inoculation (days 9, 12, 14, 16, 19, 21, 23, 26, and 28). After 5 min of centrifugation at 4 °C, 15,000 rpm, the pellet and supernatant derived from the culture broth were collected for DNA extraction and metabolites analysis, respectively (Figure 1).

### 2.4. Metabolites Analysis

The SHIME software (Prodigest, ver. 4.2.2) allowed online monitoring of the acid (0.5 M HCl) and base (0.5 M NaOH) consumption that was required to control the pH within the desired interval (6.6–6.9). Subsequently, 10 μL of each supernatant of sampled culture broth was immediately used for ammonia analysis using a Fuji Dri-chem Slide NH3-PII (Fujifilm Wako Pure Chemical Corporation) with Fuji Dri-chem (Fujifilm Wako Pure Chemical Corporation). Next, 200 μL of each supernatant was used for SCFA analysis with YMC-Pack FA (YMC Co., Ltd., Kyoto, Japan). The combined isocratic HPLC UV detector (400 nm) was operated with acetonitrile/methanol/water = 30/16/54 as the mobile phase at a flow rate of 1.2 mL/min at a temperature of 50 °C. After encoding the standard curve, each peak was integrated with the LCsolution software (Shimadzu Corporation, Kyoto, Japan, ver. 3.50).

### 2.5. Microbiome Community Analysis

DNA was extracted from the pelleted bacterial cells from a 2 mL culture broth using GENE PREP STAR PI-480 (Kurabo Industries Ltd., Osaka, Japan) with ISOSPIN Fecal DNA (Nippon Gene Co., Ltd., Tokyo, Japan). When the concentration of DNA extracted was not enough, the culture broth was increased to 4 mL. Sequencing of the 16S rRNA gene amplicon of the V3–V4 region was performed for microbial community profiling. Library preparation and sequencing were performed using an Illumina MiSeq platform with the MiSeq Reagent Kit v3 (Illumina Inc., San Diego, CA, USA) and the Nextera XT Index Kit v2 Set A (Illumina Inc.). The primers used to amplify the V3–V4 regions of the 16S rRNA gene were F (5′-TCGTCGGCAGCGTCAGATGTGTATAAGAGACAGCCTACGGGNGGCWGCAG-3′) and R (5′-GTCTCGTGGGCTCGGAGATGTGTATAAGAGACAGGACTACHVGGGTATCTAATCC-3′). QIIME2 software (2019.7 ver.) was applied for the read assembly and cleanup according to the following procedures [26]. First, the reads were assembled into contigs, followed by alignment-based quality filtering through alignment to the QIIME2-reconstructed SILVA alignment (v. 132). Upon removing any chimeras, the taxonomy was assigned, and the contigs were clustered into OTUs at 97% sequence similarity. The richest alignment of each OTU was annotated as a representative taxonomy. The results are presented as the relative abundances of the total reads.

### 2.6. Caco-2 Barrier Function Assay

The barrier function assay using Caco-2 (HTB-37, American Type Culture Collection, Manassas, VA, USA) was performed to evaluate the effect of the SHIME culture supernatant on human cells. Caco-2 cells were precultured in 20% fetal bovine serum (Biological Industries, Beit-Haemek, Israel) and 1% Penicillin-Streptomycin (Thermo Fisher Scientific, Waltham, MA, USA) added E-MEM (E-MEM with L-Glutamine, Phenol Red, Sodium Pyruvate, Nonessential Amino Acids, and 1500 mg/L Sodium Bicarbonate) for 14 days. The cell culture inserts (for a 24-well plate with 1.0 µm transparent PET membrane, Corning, NY, USA) were aseptically inserted into a 24-well companion plate (Corning), and then 2.0 × 10^3^ cells of Caco-2 were seeded in the upper part of each insert and 700 μL of E-MEM in the lower part. The plate was incubated at 37 °C in a 5% CO_2_ environment, and both parts of the inserts were changed to fresh E-MEM three times a week. Furthermore, 19 days after seeding, the Caco-2 cells formed a monolayer, an inflammatory cytokine cocktail (IC; 20 ng/mL TNF-α (R&D Systems, Minneapolis, MN, USA), 10 ng/mL IL-1β (Peprotech, Cranbury, NJ, USA) and 20 ng/mL IFN-γ (Peprotech), and sterile filtered (0.22 µm) SHIME culture supernatants (10% *v*/*v*) were added to the upper part of the inserts. Furthermore, 0 and 48 h after the addition of the culture supernatants, the transepithelial electrical resistance (TEER) value of each well was measured using an ERS-2 (Merck Millipore, Burlington, MA, USA) to evaluate the integrity of the monolayer. The experiments were performed with *n =* 3; the result is presented as the mean ± standard deviation.

### 2.7. Statistical Method

For the analysis of the microbiome community, the *p*-values for the differences between day 14 and 28 values of taxonomic relative abundances were calculated using a paired *t*-test. All the *p*-values were subsequently adjusted with a multiplicity correction to reflect a false discovery rate (0.20) using the Benjamini–Hochberg method [27]. For acid and base consumption and the Caco-2 assay, the difference was calculated using a two-way analysis of variance (two-way ANOVA) followed by Tukey’s test. For the SCFA analysis, the difference was calculated using a one-way analysis of variance (one-way ANOVA) followed by Dunnett’s test. Values of *p* < 0.05 were considered statistically significant.

## 3. Results

### 3.1. Acid/Base Consumption and Ammonia Production in the SHIME Culture

**Figure 2:**
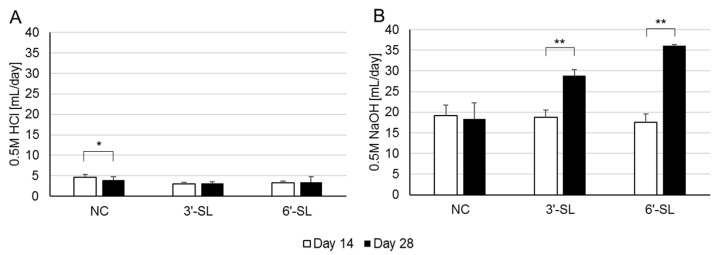
Consumption of HCl (**A**) and NaOH (**B**) during the SHIME experiment. *: *p* < 0.05, **: *p* < 0.01. NC = negative control, 3′-SL = 3′-sialyllactose, 6′-SL = 6′-sialyllactose.

In order to keep the pH at 6.6–6.9, 0.5 M HCl or 0.5 M NaOH was added to each reactor during the SHIME experiment. The average daily consumption of NaOH was significant with the addition of SL, whereas the HCl showed no difference (Figure 2). No significant differences in the ammonia concentration were observed (Figure S1).

### 3.2. SCFA Production in the SHIME Culture

**Figure 3:**
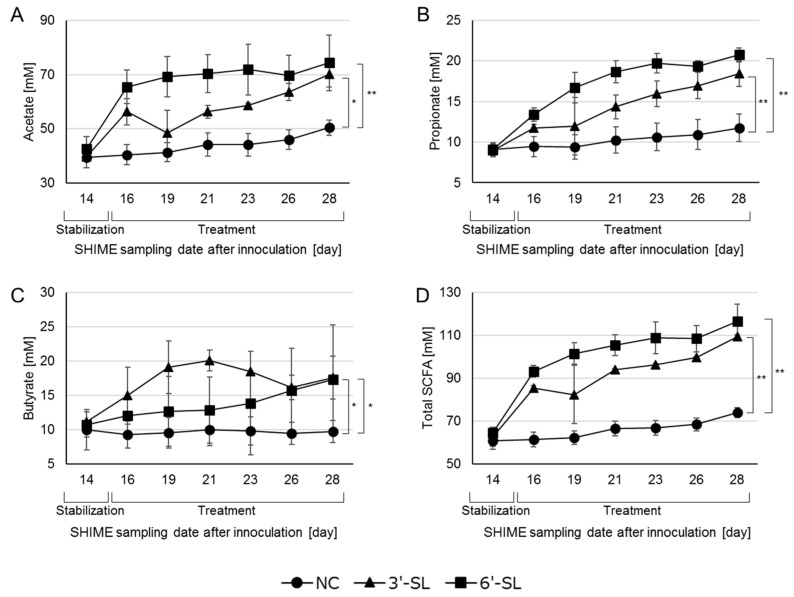
Effect of SL treatment on acetate (**A**), propionate (**B**), butyrate (**C**), and the total short-chain fatty acid (SCFA) concentration (**D**) in the SHIME culture. *: *p* < 0.05, **: *p* < 0.01. NC = negative control, 3′-SL = 3′-sialyllactose, 6′-SL = 6′-sialyllactose.

The SHIME culture supernatants collected on days 9–28 were used to quantitatively analyze the concentrations of six SCFAs (L-lactate, acetate, propionate, iso-butyrate, butyrate, and valeric acid). Since acetate, propionate, and butyrate accounted for approximately 98% of the total SCFA content, individual data for acetate, propionate, and butyrate, as well as the total SCFA content, are presented here (Figure 3). During the stabilization period, any SCFA concentration showed no differences among the groups. However, during the treatment period, the 3′-SL and 6′-SL groups showed an increase in the total production of these three SCFAs compared to the NC group. Notably, the 6′-SL group had significantly higher concentrations of both acetate and propionate compared to the NC group in a shorter period (Figure 3A,B), while the 3′-SL group significantly increased the butyrate concentration earlier (Figure 3C).

### 3.3. Change in Microbial Composition by Addition of SL

**Figure 4:**
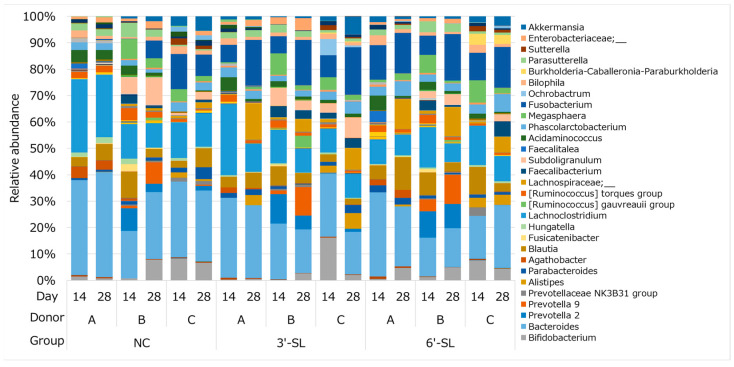
Changes in the microbial composition. Only taxa with a relative abundance of more than 2% in the SHIME culture were manually selected and presented in the usage guide. NC = negative control, 3′-SL = 3′-sialyllactose, 6′-SL = 6′-sialyllactose.

To gain insight into how SL affects the composition of the microbial community, the microbial pellets extracted from the SHIME culture on day 14 or 28 were analyzed using the 16S rRNA gene-targeted sequences. As shown in Figure 4 and Table 1, many types of genera changed during culture from day 14 to day 28, regardless of diet. When comparing the mean genus-level values of three donors, only six genera showed significant changes in the control diet, while supplementation with 3’-SL and 6′-SL caused significant changes in 19 and 17 genera, respectively. One of the genera with the highest increase in 3′-SL and 6′-SL was the butyrate-producing *Lachnospiraceae*, while the prevalence of *Lachnospiraceae* in the control group was maintained to the same extent on day 14. The prevalence of the propionate-producing *Alistipes* increased significantly in the 3′-SL but not 6′-SL, and the prevalence of the propionate-producing *Phascolarctobacterium* and butyrate-producing *Agathobacter* and *Subdoligranulum* increased significantly in the 6′-SL but not 3′-SL.

### 3.4. Effect of SL Addition into the SHIME Culture on Caco-2 Barrier Function

**Figure 5:**
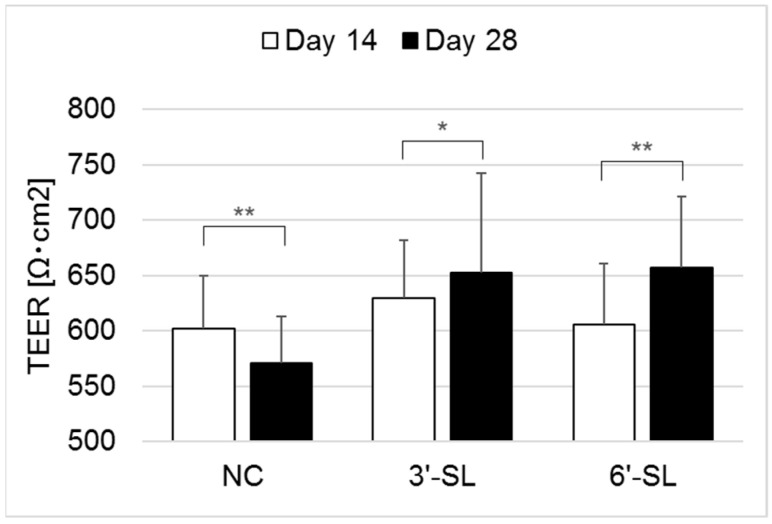
Caco-2 epithelial permeability assay. *: *p* < 0.05, **: *p* < 0.01. NC = negative control, 3′-SL = 3′-sialyllactose, 6′-SL = 6′-sialyllactose.

Caco-2 is an intestinal epithelial cell line that forms a monolayer similar to the intestinal wall, expresses proteins involved in intercellular adhesion, and forms tight junctions. Therefore, Caco-2 is commonly used as a model to evaluate the effects of bioactive components on the molecular permeability of the intestinal tract, i.e., the barrier function [28]. We examined whether the damaged barrier function of Caco-2 caused by IC could be restored by adding the SHIME culture supernatant. The culture supernatants of days 14 and 28 of each reactor were added to Caco-2 cells (*n* = 3), and the average TEER after 48 h of incubation showed that the TEER was significantly increased in the culture supernatant during the treatment period compared to the stabilization period (Figure 5).

## 4. Discussion

In the present study, we found that both 3′-SL and 6′-SL altered the microbial composition of adult humans, specifically showing an increase in the butyrate-producing *Lachnospiraceae* in both SL and the propionate-producing *Phascolarctobacterium* only in 6′-SL treatment, implying that 3′-SL appears to preferentially stimulate butyrate production. It should be noted that 3′-SL increased butyrate production compared to 6′-SL over the measurement period, while both propionate and acetate production showed opposite trends (Figure 3), partially consistent with the results concerning the SCFA production. Although, in this study, the SHIME model kept the pH in the reactors within a certain range, it is suggested that SL has the potential to regulate the gut to an acidic condition by increasing NaOH consumption (Figure 2) and promoting SCFA production (Figure 3). Furthermore, we showed that culture supernatant with each SL improves the integrity of the tight junction upon Caco-2 inflammation. Given the well-known function of butyrate to protect IECs [6], these results suggest that SL would contribute to human health through not only modulation of the microbial composition but also metabolites from the altered microbiome. However, each SL appears to act intricately in the gut. Twenty-eight types of bacterial genera were changed by 3′-SL or 6′-SL. In general, the ability of bacteria to directly or indirectly utilize sialyllactose, including its constituent sugars, is considered important for its proliferation. Given that each sialyllactose is commonly degraded into sialic acid and lactose [27], which are sugar sources for some bacteria, the results for 3′-SL and 6′-SL in Table 1 and Figure 3 are likely to be consistent. This could be explained by the intricate structure and metabolism of the two SLs. Sialidase, which can hydrolyze sialylated oligosaccharide, expressed in *E. coli*, preferentially degrades 3′-SL compared to 6′-SL [29]. Additionally, there are two kinds of sialidases: intracellular and extracellular. The latter sialidases are known to contribute to reciprocal feeding interactions with bacteria that have extracellular sialidases as first degraders and bacteria that cannot directly metabolize sialyllactose but can degrade sialic acid and lactose. These indicate that the composition of sialic acid and lactose as sugar sources for bacteria in the culture medium could differ between the SL-treated groups, resulting in differences in the bacterial proliferation profiles by each SL. Although we did not analyze the remaining HMOs and their constitutions in the culture medium, further investigations are needed to clarify the different mechanisms underlying the two SLs observed in Figure 3 and Table 1.

To the best of our knowledge, this study is the first to examine the effects of SL on the adult human fecal microbiota using the SHIME model. The gut flora are very different between infants and adults. Infants are known to have a dominant *Bifidobacterium* flora, which is believed to be supported by components contained in breast milk. In fact, some studies comparing breast milk and infant formula have confirmed such effects [30,31,32]. This bifidogenic effect has also been reported in HMOs, specifically showing in supplementations of 2′-FL and LNnT or 3′-SL in infants. Given that the effects of two types of HMOs have also been confirmed in adults, 2′-FL and LNnT may show common functions in infants and adults. However, we were unable to observe the bifidogenic effect of SL despite the presence of *Bifidobacterium*. This result was consistent with SL treatment of 24 h using another bioreactor-based gut model [33].

Only 2′FL and LNnT have been reported to affect the adult fecal or gut microbiota in in vitro models or clinical trials. Especially in infant development, *Bifidobacterium*, the main HMO-degrader in the gut microbiota, provides various benefits by colonizing the infant’s gut. Šuligoj et al. reported that the addition of 2′-FL and LNnT increased the amount of *Bifidobacterium* and acetate production in the adult fecal microbiota, the same as in infants [21]. In this study, although SL is a class of HMO, it did not show a bifidogenic effect (Table 1). Contrarily, significant increases in the propionate-producing *Phascolarctobacterium* and *Alistipes* and the butyrate-producing *Lachnospiraceae*, *Agathobacter*, and *Subdoligranulum* were observed, resulting in SCFAs increasing in the culture supernatant [34,35,36]. These results suggest that SL may have different functions in the adult gut microbiota from the roles in the gut microbiota of infants. Our findings revealed that SL did not increase *Bifidobacterium*, which is consistent with a previous study in which SL was administered to neonatal piglets [37]. Propionate and butyrate are known for their bioactivity, such as protection against cardiovascular injury, energy source for IECs, and anti-inflammatory effect by regulatory T-cell induction [10]. In an observational study of Chinese patients with benign meningiomas and malignant gliomas, the relative abundances of *Escherichia–Shigella*, *Lachnospira*, and *Agathobacter*, altered by SL in the present study, were biomarkers of brain diseases via the gut–brain axis [38]. In summary, nonbifidogenic modification of the adult gut microbiota and increases in propionate and butyrate using SL suggest the possibility of a protective effect against disease and chronic inflammatory conditions. It should be noted that SL promoted the abundance of *Fusobacterium*, which can promote malignancy by inducing DNA methyltransferases [39]. Further studies of in vitro culture experiments involving *Fusobacterium* species or species-level metagenome sequencing can reveal whether pathogenic *Fusobacterium* species are promoted by SL consumption.

Many studies suggest the beneficial effect of SCFAs on human health. Because SCFAs are mainly produced in the gut, the function of SCFAs in IECs has been studied in detail, and evidence has been accumulated in vitro and in vivo. Propionate derived from the gut microbiome reduces *Salmonella enterica* serovar. Typhimurium in mice modulates bacterial cellular function [40]. Next, using the Caco-2 model, butyrate has been reported to improve intestinal barrier function by activating AMP-activated protein kinase and enhancing the tight junctions responsible for intercellular adhesion [41,42]. In this study, the SHIME culture supernatant in which SL increased the butyrate production improved the TEER of the Caco-2 monolayer, a marker of barrier function. This is consistent with the results of previous studies and confirms that SL supplementation has a protective effect on the gut barrier function via butyrate.

This study has some limitations. The SHIME model can simulate only the human microbiome but not the human intestinal response and the interaction between microorganisms and the human intestine. Alternatively, the result of this study clearly demonstrated how SL modulates the human microbiome and the metabolite profile derived from microorganisms without considering the effects on the human intestine. The Caco-2 assay partly explained the effect of microbial metabolites on the human intestine. It seems certain that SL induces the microbial SCFA production in the gut by promoting the growth of SCFA-producing bacteria and microbial metabolites in the SHIME culture, which improves the function of the intestinal barrier. In respect of generality, the sample size of this study might not be enough to declare the prebiotic effect of SL for all adults. However, it was worth noting that the mentioned SCFA-producing bacteria and SCFA production increased consistently among all three donors. Nevertheless, the microbial community during the stabilization period differed between donors. Additional research is needed to estimate the benefits of other microbial metabolites except SCFAs and a more complicated gut model or clinical trials to confirm feasibility in humans.

In this study, we evaluated for the first time the effect of SL on the human adult fecal microbiota and human cell lines using the SHIME model. In conclusion, it is suggested that SL differs from the previously reported bifidogenic effects of HMOs, such as the increase in propionate- and butyrate-producing bacteria. Furthermore, the increased production and the protective effect on the gut epithelial cells of SCFAs were verified via the addition of the SHIME culture supernatant to Caco-2. These results indicate the potential of SL as a prebiotic with unique functions among other HMOs.

## 5. Patents

Y.S., M.K. and S.N. have filed a provisional patent concerning the use of SL for the increase of *Phascolarctobacterium*.

## Figures and Tables

**Table 1:** Effect of SL treatment on the microbial composition. Only taxa with a statistical difference in relative abundance between days 14 and 28 in the SHIME culture were manually selected and presented in the table, except for *Bifidobacterium*. The values presented are the averages of the relative abundances (%) of the three adult human donors tested (*n* = 3). Statistical differences in the relative abundance between days 14 and 28 are indicated in bold. NC = negative control, 3′-SL = 3′-sialyllactose, 6′-SL = 6′-sialyllactose.

Taxon	Group	NC		3′-SL		6′-SL	
	Day	14	28	14	28	14	28
*Bifidobacterium*		3.51	5.11	5.58	1.72	3.15	4.72
*Collinsella*		0.29	0.33	0.34	0.24	0.48	**0.22**
*Bacteroides*		27.45	30.64	25.06	**20.06**	20.93	20.43
*Odoribacter*		0.01	0.01	0.01	**0.03**	0.02	0.01
*Alistipes*		0.70	0.29	0.87	**3.28**	1.23	1.51
*Agathobacter*		1.76	**1.16**	0.74	0.23	0.75	**1.10**
*Anaerostipes*		0.14	0.12	0.10	**0.05**	0.17	**0.07**
*Dorea*		0.09	0.03	0.03	**0.00**	0.05	0.00
*Eisenbergiella*		0.05	0.03	0.03	**0.05**	0.05	0.04
*Hungatella*		1.53	0.99	0.49	**0.23**	0.43	0.38
*Lachnoclostridium*		18.09	**15.20**	16.16	9.54	13.23	**8.14**
*Lachnospiraceae* UCG-004		0.00	**0.02**	0.02	0.01	0.01	0.01
*Lachnospiraceae* UCG-010		0.00	0.00	0.01	**0.10**	0.01	**0.05**
*Tyzzerella*		0.015	0.020	0.013	**0.010**	0.026	0.011
[Ruminococcus] torques group		2.72	1.68	2.15	**1.01**	2.14	1.10
*Lachnospiraceae*;__		0.54	1.00	0.72	**8.94**	0.71	**9.40**
Candidatus Soleaferrea		0.05	0.04	0.05	**0.02**	0.08	**0.03**
*Oscillibacter*		0.21	0.12	0.14	**0.12**	0.12	0.15
*Ruminococcus* 2		0.05	**0.10**	0.04	0.02	0.14	0.02
*Subdoligranulum*		2.33	4.34	3.39	3.84	0.92	**2.24**
*Ruminococcaceae*;__		0.002	0.010	0.002	0.002	0.005	**0.000**
*Holdemania*		0.00	0.00	0.01	**0.00**	0.01	0.01
*Acidaminococcus*		2.75	1.69	2.74	1.10	3.49	**0.66**
*Phascolarctobacterium*		2.77	**2.30**	2.99	3.40	3.21	**5.08**
*Allisonella*		0.28	0.31	0.50	**0.31**	0.66	**0.27**
*Selenomonas* 3		0.00	0.00	0.00	0.07	0.00	**0.04**
*Fusobacterium*		4.54	5.11	7.13	**17.45**	10.29	**16.06**
*Bilophila*		2.05	1.34	1.96	**1.20**	2.64	**1.14**
*Ralstonia*		0.000	**0.002**	0.000	0.000	0.000	0.000
*Escherichia–Shigella*		0.81	1.26	0.99	**0.62**	0.99	**0.46**
*Proteus*		0.61	0.58	0.09	0.16	0.11	**0.41**
*Enterobacteriaceae*;__		0.65	**2.14**	1.21	**2.32**	0.69	0.80

## Data Availability

All relevant data are contained within the article and Supplementary Material.

## Supplementary Materials

The following supporting information can be downloaded at https://www.mdpi.com/article/10.3390/microorganisms12020252/s1, Figure S1: Effect of SL treatment on the ammonia concentration in the SHIME culture.
