# Supplementary material for: Sialyllactose Enhances the Short-Chain Fatty Acid Production and Barrier Function of Gut Epithelial Cells via Nonbifidogenic Modification of the Fecal Microbiome in Human Adults
Source: Microorganisms. 2024 Jan 25;12(2):252. doi: 10.3390/microorganisms12020252 (PMC10892346; doi:10.3390/microorganisms12020252)
Supplement: Supplementary file 1 [file microorganisms-12-00252-s001.zip › microorganisms-2784926-supplementary.pdf]

Sialyllactose Enhances Production of Short Chain Fatty Acid and Barrier Function of Gut Epithelial Cell by Nonbifidogenic Modification of Fecal Microbiome of Human Adults

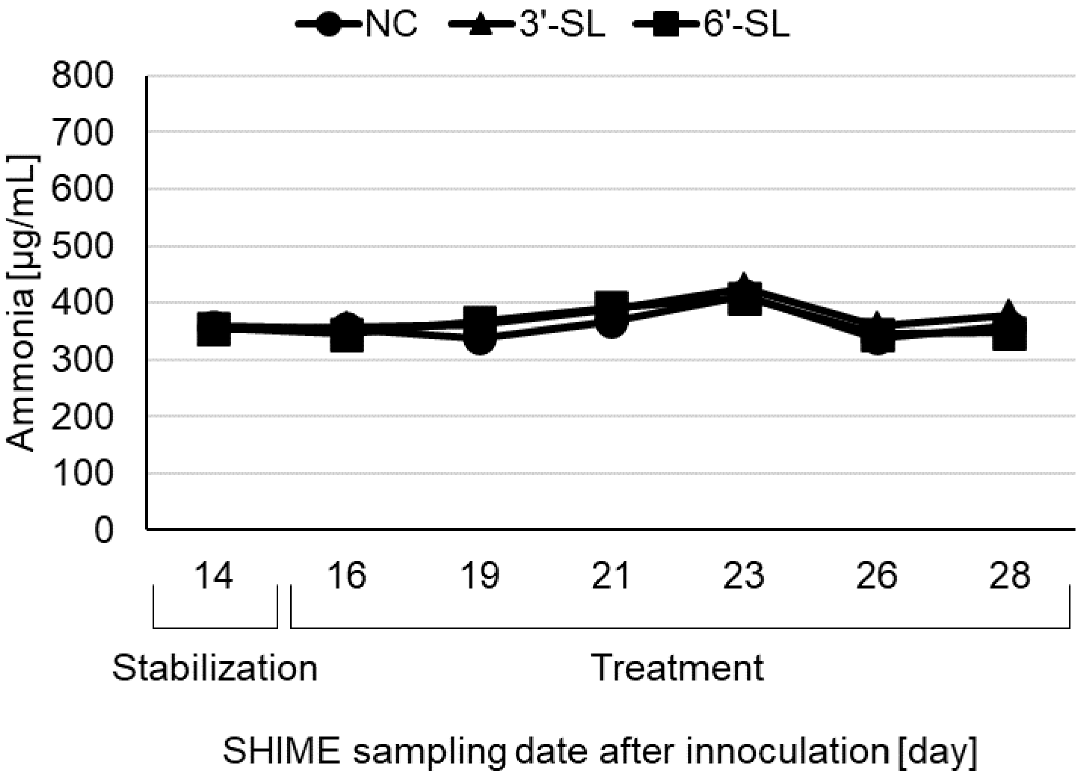

Supplementary Figure S1: Effect of SL treatment on ammonia concentration in the SHIME® culture. NC = negative control, 3′-SL = 3′-sialyllactose, 6′-SL = 6′-sialyllactose.
